# Red-Free (Green) Filter-Enhanced Gonioscopy with Smartphone: A Pilot Study

**DOI:** 10.7759/cureus.51559

**Published:** 2024-01-03

**Authors:** Md Iftekher Iqbal

**Affiliations:** 1 Glaucoma, Ispahani Islamia Eye Institute and Hospital, Dhaka, BGD; 2 Glaucoma and Cataract, Bangladesh Eye Hospital, Dhaka, BGD

**Keywords:** smartphone utilization, glaucoma, red-free filter, gonioscopy, iridocorneal angle

## Abstract

Aim: This pilot study aimed to demonstrate the usefulness of the red-free (green) filter as a novel modification for better iridocorneal angle visibility during routine gonioscopy.

Methods: As a pilot project, we observed 20 eyes of 10 patients aged 22 to 60 who attended the glaucoma department of a tertiary eye hospital in Bangladesh. All patients underwent a thorough ocular examination, from best-corrected visual acuity to the dilated fundus evaluation. Images and videos were obtained with a smartphone during gonioscopy with standard halogen light and the red-free (green) filter, subjectively analyzed by two glaucoma specialists.

Results: The mean age of the patients was 37 ± 13.42 years, of whom 70% were men. In this study, 40% of the patients had open-angle glaucoma, and 60% had open-angle without glaucoma. Without impairing the ability to see the iridocorneal angle structures in detail, the gonioscopy picture contrast was enhanced objectively for red-free filter images compared to standard light photos. The built-in warm filter of the slit-lamp also provided better visualization of the iridocorneal angle structures.

Conclusion: Using the red-free (green) filter and a warm filter instead of the traditionally used standard light of the slit-lamp may significantly enhance the diagnostic capability during routine gonioscopy.

## Introduction

Gonioscopy is an important part of modern ophthalmological evaluation, laser treatments, and minimally invasive glaucoma surgery (MIGS). It is usually utilized to distinguish between open and angle closure in clinical practice since therapy and severity vary [1,2].

It is a method for observing the iridocorneal angle (ICA), the space between the iris and the cornea through which the aqueous humor drains. The intraocular pressure (IOP) can rise if the aqueous cannot escape via the trabecular meshwork. All patients at risk for or newly diagnosed with glaucoma should have a gonioscopy performed as part of their ophthalmic screening to determine the cause of elevated IOP [2].

Good visualization of the ICA structures, especially trabecular meshwork (TM), is, therefore, essential to ensuring the proper grading of the ICA. Iridocorneal structures are more challenging to discern in patients with a minimum or lack of pigmentation, which may compromise the accuracy of the ICA grading [3].

Greenlight offers the best overall fundus image with outstanding contrast, which improves the visualization of common findings like hemorrhages, drusen, and exudates, as well as the retinal vessels. This is why fluorescein angiography is frequently performed in conjunction with the baseline green filter for "red-free" pictures [4,5]. Smartphone-based ocular imaging has recently made great strides, and it is likely to become the standard for photo documentation and follow-up in ophthalmology [6,7].

However, data on the application of a red-free (green) filter during gonioscopy is scant. Hence, a pilot study was conducted that analyses a novel technique for routine gonioscopy in which a red-free (green) filter is used to enhance the visibility of TM and other angle structures.

## Materials and methods

This was a pilot project conducted at the Ispahani Islamia Eye Institute in Dhaka, Bangladesh. The study was approved by the Institutional Review Board of the Ispahani Islamia Eye Institute (approval number: IIEI&H/IRB/2023/017). It was conducted according to the ethical guidelines outlined in the Declaration of Helsinki. Patient privacy was protected, and all patients provided their informed consent.

Patients aged 22 to 60 with glaucoma and those coming in for a regular ocular exam without any corneal pathology to ensure proper visualization of the iridocorneal angle were included. A total of 20 eyes from 10 patients with open anterior chamber angles were evaluated here, as it was conducted as a pilot project for future studies. Our institution routinely records gonioscopic procedures on video and subsequently reviews the findings of the gonioscopy. Except for the use of a red-free (green) filter, no adjustments were made to the standard gonioscopy approach.

Every individual had their best-corrected vision tested, eyes examined under a slit-lamp biomicroscope, IOP measured with a Goldmann applanation tonometer, gonioscopy with an indirect Volk 4-mirror goniolens (Volk Optical, Inc., Ohio, United States), and dilated retinal evaluation with a Volk 90D (Volk Optical, Inc.) condensing lens.

We used a non-brand universal smartphone slit-lamp adaptor here to mount a Samsung S21 Ultra 5G smartphone (Samsung Electronics Co., Ltd, Suwon-si, South Korea) with the slit-lamp ocular. Using a standard gonioscopy technique, videos and pictures of the iridocorneal angle were then taken continuously with the primary camera (108 megapixels) with an indirect Volk 4-mirror goniolens and standard halogen light (Volk Optical, Inc.). The Zeiss SL 115 Classic slit-lamp (Carl Zeiss Meditec, Jena, Germany) had a red-free (green) filter and was set to 1/4th illumination with 20x magnification. Images and videos of the ICA were taken with a warm filter as well (Video 1).

**Video 1:** Gonioscopy

**Figure 1:**
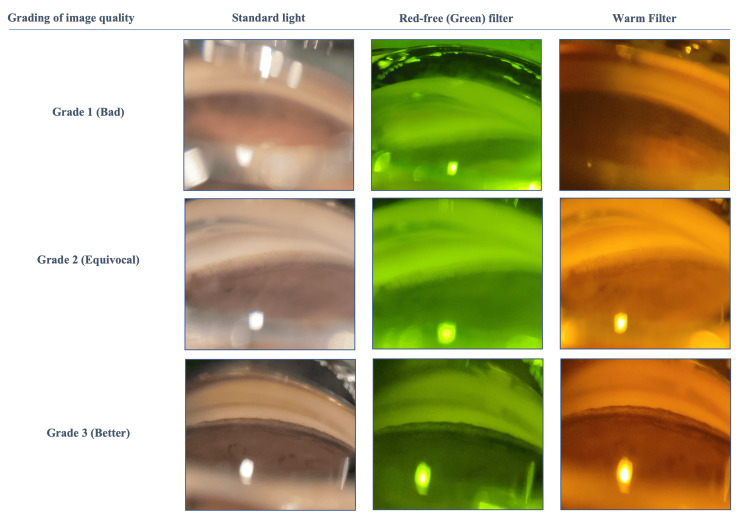
Grading of image quality

By touching the smartphone screen, each quadrant of the anterior chamber angle image or video was individually focused. Later, two glaucoma specialists subjectively analyzed those unedited images and videos regarding iridocorneal angle structures, especially the pigmentation of the trabecular meshwork on a scale of 1 to 3 against the standard light with a red-free (green) filter and a warm filter (1 = bad, 2 = equivocal, and 3 = better) (Figure 1), Shaffer grading, and other findings. If there was any disagreement between two glaucoma specialists regarding the images, then the primary observer's opinion was taken.

All the data were analyzed and percentages of the categorical variables were generated by Microsoft Excel for Mac 2021, Version 16.80 (Microsoft Corporation, Redmond, Washington, United States).

## Results

A total of 20 eyes of 10 patients were examined. The average age of the patients was 37 years (SD = 13.42), and 70% of them were male. Each individual either had open-angle glaucoma (n=4, 40%) or open-angle only (n=6, 60%).

**Figure 2:**
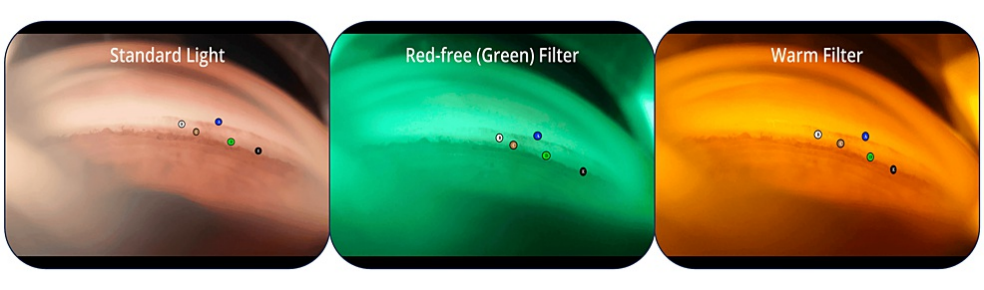
Images obtained during routine gonioscopy with a standard color, a red-free (green) filter, and a warm filter A: Schwalbe's line, B: Non-pigmented trabecular meshwork, C: Pigmented trabecular meshwork, D: Scleral spur, and E: Ciliary body band

The evaluation of the images and videos by two glaucoma specialists revealed that the red-free (green) filter provided enhanced tissue visualization for the pigmentation of the trabecular meshwork in 90% (n = 9) of the cases (Figure 2) and the quality of visualization did not get worse during regular gonioscopy.

Also, the warm color provided good contrast between the angle structures as well as being less irritating to the photosensitive patients.

Some photos and videos of 5% (n = 1) of the cases didn't have the best quality because of a number of circumstances, e.g., the slit-lamp's intense light overexposed the target area. Reducing the light intensity, using the slit-lamp's built-in diffuser, or slightly offsetting the light beam to lessen the reflection by the goniolens may improve the image quality.

## Discussion

Angle status is crucial for glaucoma management, and accurate trabecular meshwork analysis is more challenging for individuals with low or absent pigmentation. In this pilot study, two glaucoma experts objectively analyzed gonioscopic images taken during routine gonioscopy to determine the usefulness of red-free (green) filters for improving the contrast and appearance of iridocorneal structures. Using digital technology makes it possible to apply color filters during surgical procedures, resulting in improved contrast and enabling more accurate imaging of anatomical structures. According to Sandali et al., optimal digital settings improve tissue perception of Schlemm's canal and the color of the trabecular meshwork in almost 65% of instances [3]. Enhanced visibility during cataract surgery and epiretinal membrane staining has been seen by surgeons due to the utilization of digital imaging, as reported in many publications [8-11].

The implementation of cyan (greenish-blue) saturation [12] enhanced the contrast and helped identify the trabecular meshwork pigmentation. The brownish trabecular meshwork pigmentation has a greater color temperature than cyan. The color temperatures of these hues are practically opposite and, hence, they are complementary. Complementary colors have the most contrast [13]. We found that the green (red-free) filter enhanced contrast, allowing us to better see the pigmented trabecular meshwork and make proper identifications.

We also noticed that Schlemm's canal was difficult to distinguish in photos when lit at neutral color temperatures because it appeared white. However, the image turned orange if we used the warm color filter on the slit-lamp. According to the findings of Sandali et al., modifying the temperature colors can enhance the visibility of Schlemm's canal, which is typically difficult to discern using conventional color settings [3]. This modification results in a reddish look to the canal, facilitates its identification, and has the potential to enhance the precision and safety of stent placement through MIGS.

Use of a smartphone

With the introduction of smartphones, the use of portable ocular imaging devices has played a significant role in modernizing ophthalmic treatment [14]. Currently, smartphones can capture high-definition photos and videos of the entire eye, including the ocular surface, anterior and posterior segments, and angles [15,16]. Smartphones are an excellent and highly portable option for practicing ophthalmology, especially in regions where costly imaging equipment is not readily accessible. Furthermore, they offer a realistic means of relaying ocular problems and treatment options to both patients and their families [17].

**Figure 3:**
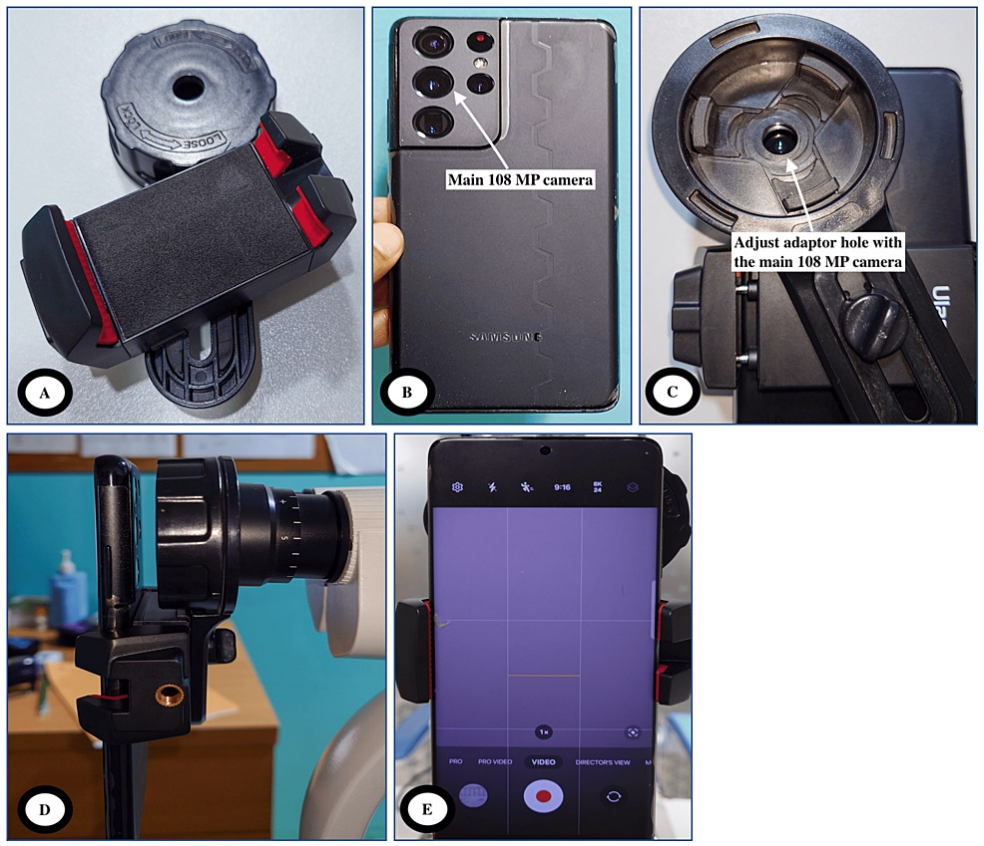
Smartphone adaptor attachment with the slit-lamp A: Non-brand universal smartphone slitlamp adaptor; B: Samsung S21 Ultra 5G showing the main 108 MP camera; C: The adaptor hole is adjusted with the camera's main 108 MP camera; D: The adaptor is mounted with a slit-lamp; E: Video mode of the smartphone's camera app is enabled.

Our study utilized a Samsung S21 Ultra 5G with a smartphone attachment to the slit-lamp (Figure 3). By touching the display of a smartphone, we were able to individually focus on the angle of the anterior chamber in each of the four quadrants. With the video mode of the phones, we were able to quickly and easily take sharp Gonio images, and we were able to save the necessary photos by simply taking screenshots. With the voice command, it was also easier to take images and videos without leaving the tasks in the hands of the examiner.

Limitations

There are several limitations in this study that need to be addressed in the future. To verify these results in a larger sample, additional research is required, and there is already an ongoing study for further evaluation both subjectively and objectively. The specific color temperatures that were used in this study should be standardized for future studies. The specific iridocorneal structures that were visualized better with a warmer color filter should be further characterized. Only superior and inferior quadrants were observed during this study, so further details are needed to evaluate all quadrants of the anterior chamber angle by documenting any findings other than open or closed angles. Also, the corneal wedge was not evaluated in this study, as only a wide-open anterior chamber angle was observed.

## Conclusions

The findings of this study suggest that the utilization of a red-free (green) filter and a warmer color filter has the potential to augment the visualization of the trabecular meshwork during standard gonioscopy procedures. This enhancement can facilitate the accurate identification of the angle structure, particularly in instances where gonioscopy interpretation is challenging and when the patient is sensitive to light. Moreover, it can be an easy modality for learning and sharing gonioscopic evaluation with smartphone recording.
